# Revealing water’s secrets: deuterium depleted water

**DOI:** 10.1186/1752-153X-7-103

**Published:** 2013-06-17

**Authors:** Vladyslav V Goncharuk, Alina A Kavitskaya, Iryna Yu Romanyukina, Oleksandr A Loboda

**Affiliations:** 1 A.V. Dumansky Institute of Colloid Chemistry and Chemistry of Water, National Academy of Sciences, Kyiv, Ukraine

**Keywords:** Deuterium depleted water, Surface tension, Viscosity, Water clusters

## Abstract

**Background:**

The anomalous properties of water have been of great interest for generations of scientists. However the impact of small amount of deuterium content which is always present in water has never been explored before. For the first time the fundamental properties of deuterium depleted (light) water at 4°C and 20°C are here presented.

**Results:**

The obtained results show the important role of the deuterium in the properties of bulk water. At 4°C the lowest value of the kinematic viscosity (1.46 mm^2^/s) has been found for 96.5 ppm D/H ratio. The significant deviation in surface tension values has been observed in deuterium depleted water samples at the both temperature regimes. The experimental data provides direct evidence that density, surface tension and viscosity anomalies of water are caused by the presence of variable concentration of deuterium which leads to the formation of water clusters of different size and quantity.

**Conclusions:**

The investigated properties of light water reveal the origin of the water anomalies. The new theoretical model of cluster formation with account of isotope effect is proposed.

## Introduction

For over thousand years water kept its secrets. At the first glance water seems to be one of the most simple and abundant substances, however in addition to H_2_O it has also H_3_O ^+^, OH ^−^, HOD, and OD ^−^ moieties [1] which are in part responsible for the multifold of unusual properties. There have been numerous attempts to explain the anomalies of water (Pauling’s, Robinson, Takahashi, Chaplin’s two states model etc.) [2-7]. The most intriguing and discussed theory on the origin of water anomalies is the water cluster model [5]. The density inhomogeneities of ∼1nm length-scale have been reported from the small-angle X-ray scattering measurements [8]. Conversely, the existence of density inhomogeneities in liquid water has been called into question recently [9]. However the attempts to interpret the x-ray diffraction data to probe the molecular arrangement even in the first coordination shell of liquid water can not provide unambiguous structural information [10-12].

At zero°C the ice structure has all hydrogens in a “bonded” state. With the gradual increase of temperature the number of broken water hydrogen bonds increases too. This process is accompanied by a decrease in the volume and an increase in the density. However, also the “competitive” process of thermal expansion should be considered, which operates in an opposite way (reduces the density). Below 4°C, at atmospheric pressure the thermal expansion coefficient (*α*=(*δ* ln *υ* / *δT*) _*P*_) is negative. The magnitude of the *α* also reflects the correlations between the entropy and volume fluctuations. These fluctuations are positively correlated at the temperature above 4°C and anti-correlated below 4°C at which decrease in volume results in an corresponding increase in entropy value. In fact, upon the formation of open hydrogen bonded network the orientational contribution to the entropy is decreased which is instantly accompanied by a volume expansion [13]. With increasing temperature from 0°C to 4°C, the effect of hydrogen bonds cleavage prevails over the thermal expansion, so the total volume decreases (large clusters break up into smaller clusters). The decrease in volume leads to an increase of density, which goes to a maximum at 4°C. At this point the density increment held due to the cleavage of hydrogen bonds is balanced by the effect of thermal expansion which causes a decrease in density. At temperatures above 4°C, the effect of thermal expansion prevails over the destruction of hydrogen bonds, resulting in the loss of the density [6,7]. Thus, one may assume that at the temperature range 0° - 4°C an increase of non-hydrogen bonded H atoms might occur due to the collapse of large supramolecular complexes into the small clusters. Reducing the size of the clusters would lead to the volume decrease which is inversely proportional to the density.

However none of the proposed models take into account isotope composition of water and therefore can not fully unravel the complexity of water. The physical properties of heavy water, water enriched with the deuterium and heavy-oxygen isotopes, are well-known [14-17]. The boiling and freezing points of such water are shifted from the relevant points of normal water. Heavy water is widely used in nuclear power reactors as a neutron moderator [18]. It has also been reported to be harmful for living beings and toxic for cells [19]. Fortunately the amount of deutereuted water molecules in normal water insignificantly small and is about 150 ppm.

The majority of science community neglects the concentration of heavy isotopes of protium in water and assumes that it consists of solely H_2_O. In this work we investigate the effect of these small quantities of D_2_O in normal water by elaborating the extreme case of the deuterium depleted water i.e. light water. The prime purpose of the present research is to study the physical properties of light water. Experimental measurements in the absence of the deuterated water indirectly tell us how important is the content of D_2_O for the magnitude of physical constants. The list of published peer-reviewed research papers on the topic of light water is scarce (less than few dozens of papers) and almost limited to the effect of deuterium-depletion on living cells [20-22]. However to the best of our knowledge the fundamental physical properties of pure light water (below 150 ppm) have never been investigated. Therefore the overall aim of the work is to generalize the obtained knowledge in order to build up a comprehensive view on the organization of water.

## Results

The density, kinematic viscosity and surface tension of water with varying amounts of deuterium obtained for the temperature regimes 4° and 20°C are shown in Table 1. The dilution of light water (2 ppm) by heavy water content up to 19 ppm elevates the kinematic viscosity and surface tension, though the density does not change significantly. This effect is observed at the both temperatures, but is more profound at 4°C. An increase in temperature leads to a significant reduction of the viscosity and surface tension values.

**Table 1:** Kinematic viscosity, surface tension and density of deuterium depleted (Superlight), Semilight, Normal, Semiheavy and Heavy water samples studied at 4°C and 20°C

**Water**	**Deuterium**	**Kinematic viscosity, mm**^**2**^**/s**		**Surface tension, mN/m**		**Density, g/cm**^**3**^	
**sample**	**content**	**4°C**	**20°C**	**4°C**	**20°C**	**4°C**	**20°C**
Superlight	2 ppm	1.5458 ± 0.0017	0.9923 ± 0.0017	76.27 ± 0.01	73.93 ± 0.01	1.0001 ± 0.00015	0.9979 ± 0.00015
	4.2 ppm	1.5179 ± 0.0017	0.9626 ± 0.0017	76.39 ± 0.01	73.93 ± 0.01	0.99950 ± 0.00015	0.9982 ± 0.00015
Semilight	18.9 ppm	1.5145 ± 0.0017	n/a	77.58 ± 0.01	n/a	0.99995 ± 0.00015	0.9999 ± 0.00015
	37 ppm	1.4863 ± 0.0017	n/a	77.58 ± 0.01	n/a	0.99980 ± 0.00015	n/a
	41.3 ppm	1.4900 ± 0.0017	n/a	77.34 ± 0.01	n/a	0.99989 ± 0.00015	0.9999 ± 0.00015
	96.5 ppm	1.4627 ± 0.0017	n/a	77.58 ± 0.01	n/a	1.00040 ± 0.00015	1.0004 ± 0.00015
Normal	144.7 ppm	1.5746 ± 0.0017	1.0075 ± 0.0017	75.49 ± 0.01	72.58 ± 0.01	1.00027 ± 0.00015	0.9975 ± 0.00015
	145.6 ppm	1.5896 ± 0.0017	1.0057 ± 0.0017	74.87 ± 0.01	72.75 ± 0.01	1.00030 ± 0.00015	1.0001 ± 0.00015
Semiheavy	52%	1.6502 ± 0.0017	1.0534 ± 0.0017	74.83 ± 0.01	70.39 ± 0.01	n/a	n/a
	53%	1.6514 ± 0.0017	1.0862 ± 0.0017	74.87 ± 0.01	70.39 ± 0.01	1.05850 ± 0.00015	1.0532 ± 0.00015
Heavy	90.98%	1.7813 ± 0.0017	1.1968 ± 0.0017	70.08 ± 0.01	67.08 ± 0.01	1.1052 ± 0.00015	1.1052 ± 0.00015
	99.96%	1.7500 ± 0.0017	1.274 ± 0.0017	69.93 ± 0.01	67.80 ± 0.01	1.1040 ± 0.00015	1.1042 ± 0.00015

It is interesting to note how the small variations of D_2_O molecules (for example from 4 to 19 ppm) can affect the physical properties of the whole system. This effect becomes more pronounced as the temperature lowers, but diminishes or masked by other collective processes at >150 ppm and under conditions of the higher temperatures. With the increase of deuterated water molecules in the range of 19-97 ppm the kinematic viscosity decreases from 1.5145 to 1.4627 mm^2^/s, while the surface tension is almost constant (77.58) at 4°C. The densities of the water samples with deuterium contents ranging from 2 to 97 ppm do not deviate at both temperature values 4°C and 20°C. Substantial deviations of the surface tension and kinematic viscosity are observed in water probes with a D/H ratio of 146 ppm. At 4°C the kinematic viscosity sharply increases and constitutes 1.5896 mm^2^/s. The surface tension’s decrease pattern pertains to water at 4° and 20°C as well, though it is more characteristic at 4°C. The deuterium concentration less than 2 ppm has not be achieved experimentally to date.

## Discussion

As is expected the density of light water does not show any appreciable change due to the very small quantity of deuteriated water at the 1 - 150 ppm level. Surface tension (ST) on the other hand is the result of weak inter-molecular interactions in liquid water. The decrease of ST with increasing temperature is usually associated with a decrease in density due to the increase of intermolecular distances. However, it should be noted, all experimental measurements indicate that the changes of ST far exceed the corresponding change in density. For example, in the temperature range from 0 to 100°C, the volume is changed by 4% while ST is reduced by 22%. This significant change of ST can not be explained only by the change in the density or the interaction of individual molecules, but can be tackled on the basis of the cluster model. With increasing temperature, the average cluster size decreases. Also, the increase in temperature enlarges the intercluster distances, which entails a reduction of unbound O-H groups in the surface layer, that causes a decrease of ST.

At a constant isotopic content the size of the clusters depends on the temperature. The gradual increase of the temperature also leads to an increase of intercluster distances and the breaking of large clusters into the smaller ones which reflects an increase of free hydrogen bond O-H groups. However at selected constant temperatures, the fraction of NHB H atoms varies as a function of the concentration of deuterium, which is manifested in a change in the characteristics of the viscosity and the surface tension. This change in the physicochemical properties of water means that the deuterium introduced into the system affect the distribution of the bound and unbound OH groups. The question arises is how? From the data conducted for experiments with a light water one sees that the surface tension has a maximum value for a content of deuterium ≤ 96.5 ppm. The atoms of deuterium having mass twice of the protium make more stronger O-D bonds than O-H bonds. Our theoretical calculations show stable spherical structures with encapsulated guest molecules [23]. We anticipate that the deuterium atoms form the core of the cluster, around which the light molecules are arranged symmetrically in a strictly defined mosaic order. Upon reduced concentration of deuterium (≤155 ppm) the clusters can be expected of a larger size than in extreme cases of pure light and heavy water, which means fewer NHB H atoms and hence a greater surface tension. Because the larger clusters have a lower “mobility” in comparison with small water clusters, then the dynamic viscosity must also to increase with increasing cluster size and the registered deviations of kinematic viscosity values must be in consistency with the corresponding deviations of dynamic viscosity at the condition of constant density of water.

**Figure 1:**
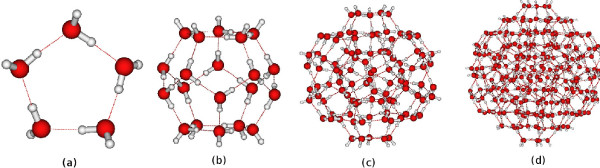
**Pentamer coordinated water network: (a)** cyclic pentamer, **(b)** 5^**12 **^**dodecahedral cluster (H**_**2**_**O)**_**20**_**,(c)** homological icosahedral (H_**2**_**O)**_**100 **_**and(d)(H**_**2**_**O)**_**280 **_**clusters.**

Dilution of the light water with the heavy water content should lead to the increase of the cluster size. This trend reaches its maximum at D/H ∼150 ppm inherent to the normal water. Then, however, the further deuterium atoms compete with each other in the process of cluster formation, which leads to a decrease of the clusters size under the excess of the deuterium isotope. Let’s try to answer the question what is really behind this mechanism? In our earlier work [23] we investigated the homologous series of icosahedral water clusters with various inclusions (CO_2_, CH_4_, (H_2_O) _*n*_) (see Figure 1). Among these series the smallest spherical cluster, which can accommodate, for instance a deuterated hydronium cation, is dodekahedra (b) with 20 water molecules composed from 12 pentamers (a). Next to it is a cluster (c) consisting of 100 water molecules on the surface of which there are 30 unbound hydrogen atoms. If 4 additional deuterated active centers appear then the cluster can break down into five basic dodecahedra, which together will produce 50 unbound hydrogen atoms (NHB H atoms). The next largest cluster (d) contains 280 water molecules and only 60 NHB H atoms. The collapse of such a cluster into 14 elementary subclusters will make possible the formation of 140 NHB H atoms. Thus an increase in deuterium content leads to a decrease in the size of the clusters, which in its turn leads to an increase of NHBs, and the last in our opinion, causes a decrease of surface tension and viscosity under conditions of constant temperature.

When the ice melts at 0°C into the liquid water it absorbs an energy of 80cal/g [24]. This energy 80cal/gm does not lead to an increase in temperature. We may speculate that the major part of this energy is spent for cleavage of 13% of the hydrogen bonds in the structure of ice, while the 87% of the bonds may remain intact. Therefore, the cluster sizes must be huge in order to accommodate 87% hydrogen bonds “inherited” from the structure of ice. Small clusters mean a large number of broken hydrogen bonds. The experimental data display the decrease in the size of the clusters with the increase in temperature [25]. Also theoretical calculations [26,27] show the broadening of oxygen-oxygen radial distribution function upon temperature increase. Apparently the elongation of inter-cluster distance contributes to the water density decrease. The increase in inter-cluster distances with increasing temperature also leads to a decrease in the number of NHB H atoms per unit area and, consequently, ST reduction.

Finally, considering the role of isotope effect on physical properties of water one can not neglect the O^18^/O^16^ ratio. Based on results obtained from our investigation we may conclude that the concentration of the heavy isotope of oxygen is closely related with the concentration of deuterium. The increase of deuterium connected with the increase of ^18^O/^16^O ratio: at 4 ppm of D/H the O^18^/O^16^ is 910 ppm,; at 52,53% D/H the ^18^O/^16^O concentrations are 1479,1552 ppm correspondingly. This indirectly signifies the bound character of these two heavy isotopes. The simultaneous increase or decrease of ^18^O and ^2^H quantities become possible during formation of covalent bonds between them which give appearance to the D_2_^18^O, HD^18^O molecules. It should be noted that these molecules make hydrogen bonding stronger than the ^1^H_2_^16^O or HD^16^O species due to the lower energy of zero point vibrational level. In our opinion the heavy isotopes of oxygen when they participate in cluster formation play a static role and make an additional contribution to the stability of the cluster while the deuterium atoms govern the whole process of water structuring and hence causes the qualitative changes of physicochemical properties of water.

For the first time the experimental studies have been conducted on deuterium depleted water. The anomalous properties of water at 4°C are found to be due to the heavy isotope of protium, which is responsible for the water cluster formation. The developed conception of determining factor of deuterium is in a good agreement with the experimental data obtained for the water samples with various deuterium content at the same conditions. The represented model provides a comprehensive assessment of the “isotope composition - water structure” relations.

## Materials and methods

Water samples with the D/H ratio 2.0; 4.2; 18.9; 37.0; 96.5; 144.7; 145.615 ppm have been obtained by vacuum distillation and purchased from Clarte company (Moscow, Russia). The heavy water 90.98% has been produced by “Cinta Crystal” Ltd. (Kyiv, Ukriane) in accordance with the standard specification (TU) 95-1893-89; the 99.96% heavy water purchased from MERCK company (Darmstadt Germany). The water samples with the 52% and 53% content of deuterium have been obtained by dilution of 90.8% heavy water using light water (4.2 ppm). The semiheavy water (52, 53% D/H) has been studied due to the maximum content of HDO molecules formed in the self exchange equilibrium reaction H_2_O+D_2_O ⇔ 2HDO. Analysis of the isotopic composition of water has been performed using modified mass-spectrometer (MI-1201) at the Institute of Geochemistry of the Environment, National Academy of Sciences.

The modified stalagmometric method is used for the measuring surface tension. The surface tension is calculated according to the formula:

(1) $$\sigma =\frac{\text{Vg}}{2\mathrm{\pi R}}(\frac{\rho }{n})$$

where volume *V* corresponds to *n* drops, which are released from the stalagmometer with the radius of stalagmometer tube R, *ρ* is the density of the liquid. In order to exclude the factor of the stalagmometer dependence, the drops number of standard (n _*x*_) and investigated (n _*o*_) liquids is counted. If the liquid with known surface tension is used then the surface tension of the other liquid can be calculated from the equation:

(2) $$\sigma_{x}=\sigma_{o}\frac{n_{o}\rho_{x}}{n_{x}\rho_{o}}$$

Methodology for the obtaining density, kinematic viscosity parameters can be found elsewhere [28]. The measurements are carried out in a sealed box, maintaining the temperature of 4 ± 0.1°, 20 ± 0.05°at a relative humidity of 60% and a pressure of 750 mmHg. All experiments are carried out above the dew point.

The relative mean errors are 0.11% (kinematic viscosity), 0.01% (surface tension) and 0.015% (density).

## Competing interests

The authors declare no competing interests.

## Authors’ contributions

VG designed and supervised the project; AK and IR carried out the experiments; VG, AK and OL analyzed the data; OL simulated the model and wrote the manuscript. All authors discussed the results and commented on the manuscript. All authors read and approved the final manuscript.
